# *Papaver somniferum* in seventeenth century (Italy): archaeotoxicological study on brain and bone samples in patients from a hospital in Milan

**DOI:** 10.1038/s41598-023-27953-1

**Published:** 2023-02-28

**Authors:** Gaia Giordano, Mirko Mattia, Lucie Biehler-Gomez, Michele Boracchi, Stefania Tritella, Emanuela Maderna, Alessandro Porro, Massimiliano Marco Corsi Romanelli, Antonia Francesca Franchini, Paolo Maria Galimberti, Fabrizio Slavazzi, Francesco Sardanelli, Domenico Di Candia, Cristina Cattaneo

**Affiliations:** 1 Department of Biomedical Science for Health, University of Milan, Milan, Italy; 2 Bureau of Legal Medicine and Insurance, Department of Biomedical Science for Health, University of Milan, 20133 Milan, Italy; 3 LABANOF (Laboratorio di Antropologia e Odontologia Forense), Department of Biomedical Science for Health, University of Milan, 20133 Milan, Italy; 4 Unit of Radiology, IRCCS Policlinico San Donato, Via Morandi 30, San Donato Milanese, 20097 Milan, Italy; 5 Neurology 5/Neuropathology Unit, Fondazione IRCCS Istituto Neurologico C. Besta, 20133 Milan, Italy; 6 Department of Clinical and Community Sciences, University of Milan, 20122 Milan, Italy; 7 Service of Laboratory Medicine1-Clinical Pathology, IRCCS Policlinico San Donato, San Donato Milanese, 20097 Milan, Italy; 8 Foundation IRCCS Ca’ Granda Ospedale Maggiore Policlinico di Milano, 20122 Milan, Italy; 9 Department of Cultural and Environmental Heritage, University of Milan, Via Festa del Perdono 7, 20122 Milan, Italy

**Keywords:** Mass spectrometry, Liquid chromatography

## Abstract

In this paper, we present the results of toxicological analyses of preserved brain tissue and bone samples from the remains of the seventeenth century patients of the *Ospedale Maggiore*, the main hospital in Milan and one of the most innovative hospitals in Europe from the Renaissance period. Beneath it, the crypt functioned as the burial place for the deceased of the hospital. In this multidisciplinary study of the remains, toxicological analyses in particular were performed with HPLC–MS/MS on different biological samples from nine individuals. Anthropological, paleopathological, histological, radiological examinations and radiocarbon dating were also carried out. As a result, archeotoxicological analyses revealed the presence of codeine, morphine, noscapine and papaverine, derived from *Papaver somniferum*, a plant present in the hospital pharmacopeia used as a narcotic, analgesic, astringent, coagulant, and antitussive agent. Such analyses have shed light on the pharmacological therapies administered to the patients near the time of death and have implemented our knowledge of medical treatment and drug administration in the 1600’s.

## Introduction

At the turn of the Renaissance and the Modern Age, Milan acquired more importance in the field of medicine with the institution of the first hospital of Europe with free medical assistance to the poor: the *Ospedale Maggiore*, called *Ca’ Granda*^1^.

In 1456, Francesco Sforza, duke of Milan, founded the *Ospedale Maggiore* in the heart of the city, a major healthcare institution dedicated to the care of the poor social class of Milan. It became the main hospital of the city in the sixteenth century and a model during the Renaissance for similar institutions across Italy, and even Europe, because of its innovative and avant-garde free medical assistance to the poor, care of the ill and hospital organization. An improvement of the efficiency in healthcare was established by specialized therapies instead of general assistance, by employing specialized doctors and surgeons rather than generic nurses, by introducing daily medical rounds and by reforming hygienic conditions through the installation of an efficient sewage system and daily change of linens of the beds of the patients^1,2^.

Annex to the hospital, the crypt of the Church of the *Beata Vergine Annunziata* was used as place of burial for the deceased patients of the Ca’ Granda for almost the entire span of the seventeenth century. Different chambers, created for the deposition of the bodies, were built under the floor of the crypt; inside these 14 chambers lie an estimated 2.9 million bones or over 10,000 individuals^1^.

Another extraordinary aspect of this context is the presence of historical archives, still preserved, concerning the administration and organization of the hospital, patient care and the pharmacopoeia (drugs) used in the hospital to treat the patients’ diseases. The latter was an Austrian pharmacopeia readapted for Italian use reporting all plants and medical preparations (mixture of plants and animal derivatives) produced in the pharmacy of the hospital from the fifteenth century to the last days of the hospital in the early twentieth century. Moreover, inside the pharmacopeia are listed all the medical plants as well as therapeutic preparations used to cure the patients; furthermore, the part of the plant utilized (i.e., seed, grass, bud, flower, leave, resin, root) and the type of preparation (i.e., balm, extract, oil, ointment, pill, powder, syrup) were specified.

Analytical and forensic toxicology when applied to ancient remains strengthen the field of archeotoxicology, allowing the detection of molecules present inside the human remains, thus implementing our knowledge on the history of medicine and disease as well as on habits and even environmental pollution. In this perspective the archaeological context of the crypt of *Ca’ Granda* in Milan offers an extraordinary substrate on which to verify the presence in the human remains of drug therapies mentioned in the archives of the hospital (or unexpected ones), but never extracted from skeletal remains, as well as the opportunity to test to what extent modern toxicological techniques can help in the reconstruction of the past.

Indeed, the greatest challenge in archaeotoxicology is that it deals with complex and unconventional biological matrices that have been subjected to decomposition, diagenesis, taphonomic processes and very long post-mortem intervals (PMI). It is thus essential to choose the most adapted biological matrices able to preserve molecules of toxicological relevance. With this in mind, in this study, we decided to select biological matrices possibly more prone to survive decomposition, and “resist” diagenesis and taphonomic processes in general as well as possibly being more prone to preserving the analytes contained inside—specifically, bone and mummified brain tissue. The brain tissue is an organ often collected for toxicological investigation^3^ that reflects the drugs present in the circulatory stream of a body at the time of death of a subject. The analytes can pass the blood–brain barrier remaining trapped inside the brain tissue at the time of death of an individual^3^. Therefore, preserved brain tissue of the Ca’ Granda crypt that underwent a saponification process (an abiotic transformative and conservative process that develops adipocere, an insoluble soap that maintains and preserves organs) which has been preserved and maintained up to present days^4^ seemed a promising substrate.

Even the bone tissue constitutes an alternative biological matrix that can be used in archaeotoxicology for analytical investigation, considering that it is one of the only remaining biological matrices that can be found after a very long PMI^5^. The arteries that enter, through the nutritive foramina, inside the bone tissue, transport the analytes in this matrix. The bone tissue then incorporates the molecules into the inorganic matrix through the bone remodeling cycle^6^, hence a fraction of these substances remains trapped inside the inorganic portion and can be detected after the death of the individual even after a very long PMI^5^. Therefore, the bone tissue at times acts as shield for the analytes of toxicological interest preserving the molecules inside its inorganic portion until the time of analyses^5,7–9^.

As a consequence, the present study also aimed at a specific choice of biological samples and customized extraction procedures and chromatographic techniques which could reduce the limits imposed by diagenesis and taphonomy.

In literature, many studies have shown that medication drugs and drugs of abuse can be detected in preserved tissues, hair and bones^10–12^ and how to determine their presence in bones^5,7,8,10,17–20^, nails^21–23^, teeth^10,24–26^ and hair^10,12–16,21^. In these studies, the detection of substances was limited to some drug categories, like antidepressants^7,17,20,21^, antipsychotics^7,17,20^, amphetamine^7^ and methamphetamines^5^, benzodiazepines^5,7,18,20^, cannabinoids^5,7,10–12,26^, opioids^5,7,8,18–20,22–25^, non-benzodiazepine, sedative hypnotic^20^ and stimulants^5,7,10–16,18,20,22^, either in single individuals^8,11,12,17,23,25^, or small populations^5,7,10,18–22,24,26^. The historic period from which the biological samples were collected varies from Copper Age (6000 BC–3000 BC) with mummies^10^, Antiquity (3000 BC–476 AD)^10,12,14,15^, Middle Ages (476 AD–1492 AD)^10,13,15,16^, and Contemporary Era (1789 AD–ongoing)^5,7,8,17–26^.

Methods used for toxicological investigation have been primarily a GC–MS analyzer^10,12–14,16–19,22–26^ and RIA^10,12,15,16^, followed by a more recent instrumentation, the LC–MS analyzer^5,7,20,21^. In one case, the biological samples were analyzed with fluorescence polarization immunoassay and gas chromatography-flame ionization detector^8^. The literature review has been summarized in Table 1.

**Table 1:** Summary of data obtained from literature review.

References	Time period	Population of interest	Sample size	Biological matrices	Methodology	Toxicological findings
^10^	5000 BC-1500 AD	Peruvian, Egyptian, Sudan and German mummies	95	Bone, hair, teeth and soft tissues	L/L extraction RIA and GC–MS	Cannabinoids, stimulants
^11^	circa 950 BC	Egyptian mummies	–	Soft tissues	–	Cannabinoids
^12^	circa 950 BC	Egyptian mummy	1	Hair, soft tissues	L/L extraction RIA and GC–MS	Cannabinoids, stimulants
^13^	circa 1000 AD	Peruvian mummies	7	Hair	GC–MS	Stimulants
^14^	circa 2000 BC	Chilean mummies	163	Hair	L/L extraction RIA and GC–MS	Stimulants
^15^	2000 BC-1500 AD	Chilean mummies	8	Hair	L/L extraction RIA and GC–MS	Stimulants
^16^	800–1350 AD	Skeletons	86	Hair	RIA and GC–MS	Stimulants
^17^	Contemporary/forensic	Cadaver	1	Heart blood, femoral blood, urine, liver, lung, bone	L/L extraction GC–MS	Antidepressants, antipsychotics
^18^	Contemporary/forensic	Cadaver	39	Bone	L/L extraction GC–MS	Opioids, stimulants, benzodiazepines, basic drugs
^7^	Contemporary/forensic	Cadaver	6	Bone, bone marrow	L/L extraction LC–MS	Antidepressants, antipsychotics, amphetamines, benzodiazepines, cannabinoids, opioids, stimulants
^8^	Contemporary/forensic	Cadaver	1	Bone, bone marrow	L/L extraction FPI and GC-FID	Opioids
^19^	Contemporary/forensic	Cadaver	3	Bone	SPE extraction GC–MS	Opioids
^20^	Contemporary/forensic	Cadaver	30	Bone	ASE extraction LC–MS	Benzodiazepines, stimulants, antidepressants, antipsychotics, anesthetics, opioids, non-benzodiazepine, sedative hypnotic
^5^	PMI 23–29 years	Skeletons	7	Bone	ASE extraction LC–MS	Benzodiazepines, opioids, methamphetamines, stimulants, cannabinoids
^21^	Contemporary/forensic	Cadaver	–	Hair, nails	LC–MS	Antidepressants
^22^	Contemporary/forensic	Cadaver	6	Nails	SPE extraction GC–MS	Stimulants
^23^	Contemporary/forensic	Cadaver	10	Nails	L/L extraction SPE extraction CG-MS	Opioids
^24^	Contemporary/forensic	Cadaver	24	Teeth	L/L extraction GC–MS	Opioids
^25^	Contemporary/forensic	Cadaver	1	Hair, teeth	L/L extraction SPE extraction GC–MS	Opioids
^26^	Contemporary/forensic	Living individuals	29	Teeth	SPE extraction GC–MS	Opioids, cannabinoids, stimulants

*L/L extraction* liquid/liquid extraction, *RIA* radioimmunoassay, *GC–MS* gas chromatography–mass spectrometry, *LC–MS* liquid chromatography–mass spectrometry, *FPI* fluorescence polarization immunoassay, *GC-FID* gas chromatography-flame ionizer detector, *SPE extraction* solid phase extraction, *ASE extraction* accelerated solvent extraction.

In this perspective, the purpose of this study was to investigate nine samples of preserved brain tissue and corresponding eight crania (in one case a brain fragment was found free of its cranium), with the specific aim of highlighting the pharmacological therapies administered to the patients by the specialized doctors of the hospital through toxicological analyses with high-performance liquid-chromatography–tandem mass spectrometry (HPLC–MS/MS) analyses on preserved brain tissues and bone samples. To the best of our knowledge, there are no reports of opioids detected in biological samples with a post-mortem interval (PMI) of over 25 years or reports that compare the toxicological results with an extremely detailed pharmacopeia. Therefore, the present archeotoxicological study is the first to search (and report) active molecules from *Papaver somniferum* on ancient human remains and, in particular, on bone. It also is the first to associate substances reported in hospital records and pharmacological therapy to the remains of patients.

## Results

### Anthropological investigations

All biological information obtained from anthropological and paleopathological investigations are reported in Table 2.

**Table 2:** Results of anthropological analyses performed on samples under investigation (X = performed, “*–*” = absent).

Original sample n°	Sample no	Sex	Age (years)	Radiological imaging		Pathological signs	Trauma	Population affinity	Stature	Toxicological results
				X-rays	CT					
MI CG 21-O-US4-1000	PBT1	Male	ND	X	–	–	–	–	–	Noscapine
	C1									–
MI CG 21-O-US4-I39	PBT2	Male	30–45	X	X	Tertiary syphilis on right parietal bone: *caries sicca* (stages 1–3)	–	–	165.9 ± 4.05 cm	–
	C2									–
MI CG 21-O-US4-1002	PBT3	Female	Young adult	X	–	–	–	White	–	–
	C3									Codeine, morphine, papaverine
MI CG 21-O-US4-155	PBT4	Female	ND	–	–	–	–	–	–	–
	C4									Morphine, papaverine
MI CG 21-O-US4-1003	PBT5	Female	Young adult	X	X	–	Ante-mortem trauma on left parietal bone	White	–	Codeine
	C5									–
MI CG 21-O-US4-1004	PBT6	ND	ND	X	–	–	–	–	–	–
	C6									–
MI CG 21-O-US4-1005	PBT7	ND	11–12	X	–	–	–	–	–	Papaverine
	C7									–
MI CG 21-O-US4-1006	PBT8	Male	ND	X	–	–	–	–	–	–
	C8									–
Brain	PBT 9	–	–	–	–	–	–	–	–	Papaverine

The crania were all fragmented, mostly constituted of bones of the neurocranium, except for C3 and C5 which are almost complete with only part of the parietal and occipital bones missing, respectively. The bones were fragile, prone to fragmentation, and often covered with a white staining.

Due to the absence of cranium in case no. 9, the biological profile was performed only in eight cases resulting in three males (C1, C2, C8) and three females (C3, C4, C5), based on Walker^27^ and Buikstra and Ubelaker^28^. For two crania (C6, C7), the determination of sex was not possible due to the taphonomic absence of the areas of the cranium needed for the analysis of the morphometric traits. For similar reasons, in four cases, the estimation of age-at-death could not be performed (C1, C4, C6, C8). In the remaining four crania, the age was estimated^29–31^ and revealed two young adults (C3, C5) in which age could not be further detailed because of fragmentation, a 30–45 years old individual (C2) based on palatine sutures^31^, one subadult (11–12 years old, C7) based on AlQahtani 2019^30^. Population affinity could be estimated in two crania (C4, C6) of European White ancestry through Hefner morphoscopic traits^32,33^.

**Figure 1:**
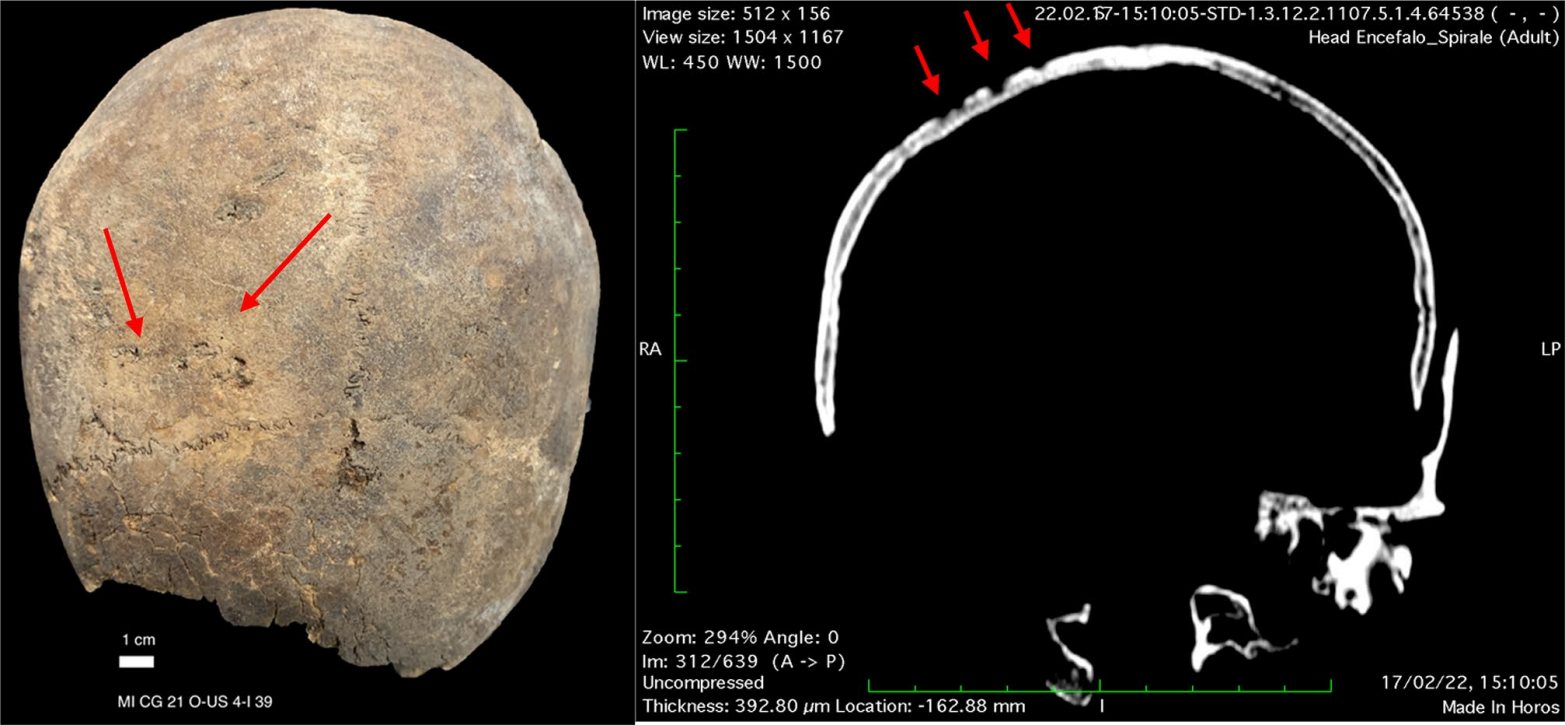
Signs of tertiary syphilis on cranium MI CG 21 O-US 4-I 39 (red arrow), both photographs and CT image.

**Figure 2:**
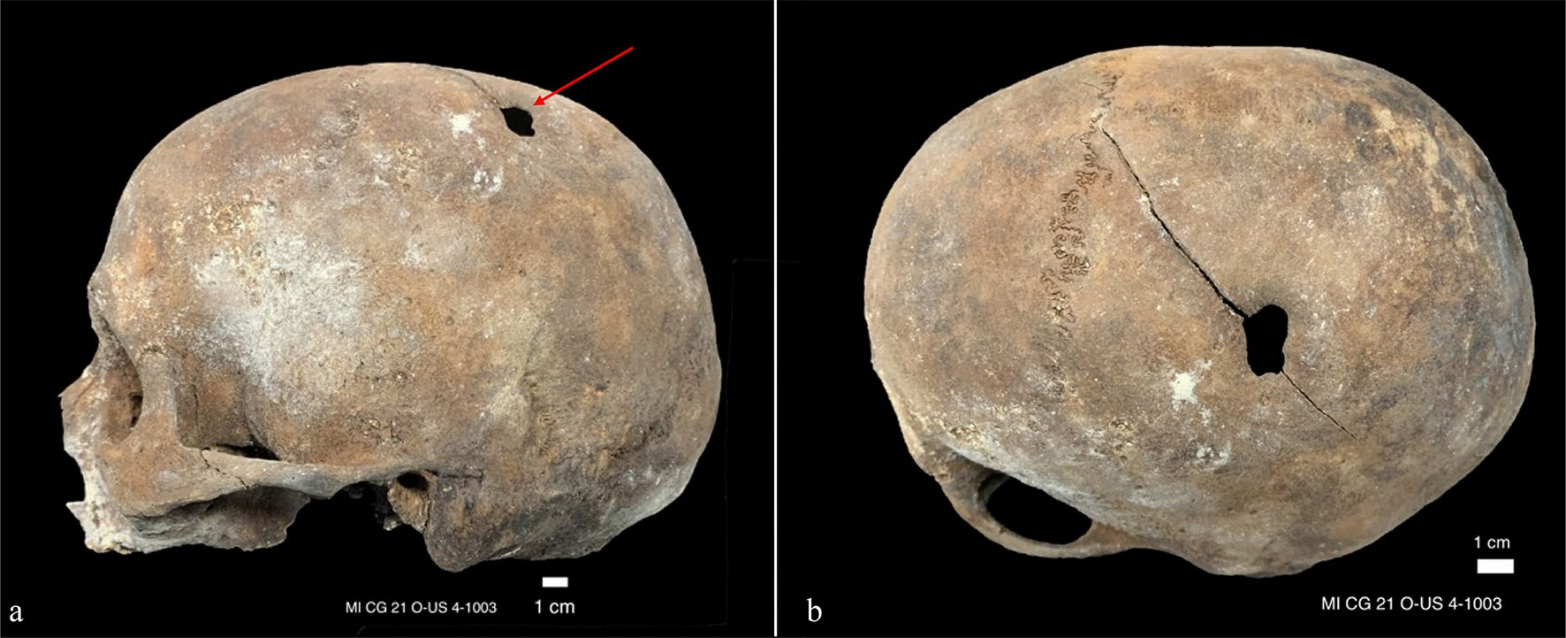
(**a**) Left lateral view of cranium MI CG 21 O-US 4-1003. Ante-mortem trauma of the left parietal bone (arrow), followed probably by trepanation and long term healing; (**b**) superior-lateral view of ante-mortem trauma on cranium MI CG 21 O-US 4-1003.

In spite of the commingling of the remains, C2 was anatomically associated to other bones (right clavicle, right humerus and eight right ribs). Based on humeral bone length, stature was estimated at 165.9 ± 4.05 cm (mean ± standard deviation). The cranium presented pathological signs of tertiary syphilis confirmed by CT analysis, with localized porosity and bone cavitation involving the outer table^34,35^ (Fig. 1). Moreover, antemortem trauma on the left parietal bone was reported in C5 which could be the result of either sharp force trauma or trepanation following blunt force trauma. The lesion, 16.0 × 14.1 mm, appeared as an area of bone loss of an oval shape with a depression on all sides (0.64 mm) and remodeling of margins; two minor fracture lines diverge from this complex. Although some forensic cases of survival to sharp force injury to the head, where a blow has caused the loss of bone tissue and secondary radiating fracture lines, exist, a different and perhaps more solid interpretation would be that of a linear fracture of the left parietal bone through blunt force trauma then treated with a scraping trepanation followed by long term healing (Fig. 2).

### Radiocarbon investigation

Radiocarbon dating was performed on the bone sample collected from cranium C2 belonging to stratigraphic unit 4 of Chamber O of Ca’ Granda crypt confirming the usage of the crypt in the seventeenth century. Indeed, the calibrated results reported with 95.4% of probability: (55.4%) 1539–1635 cal AD (411–351 cal BP) or (40%) 1460–1540 cal AD (490–420 cal BP).

### Radiological imaging

**Figure 3:**
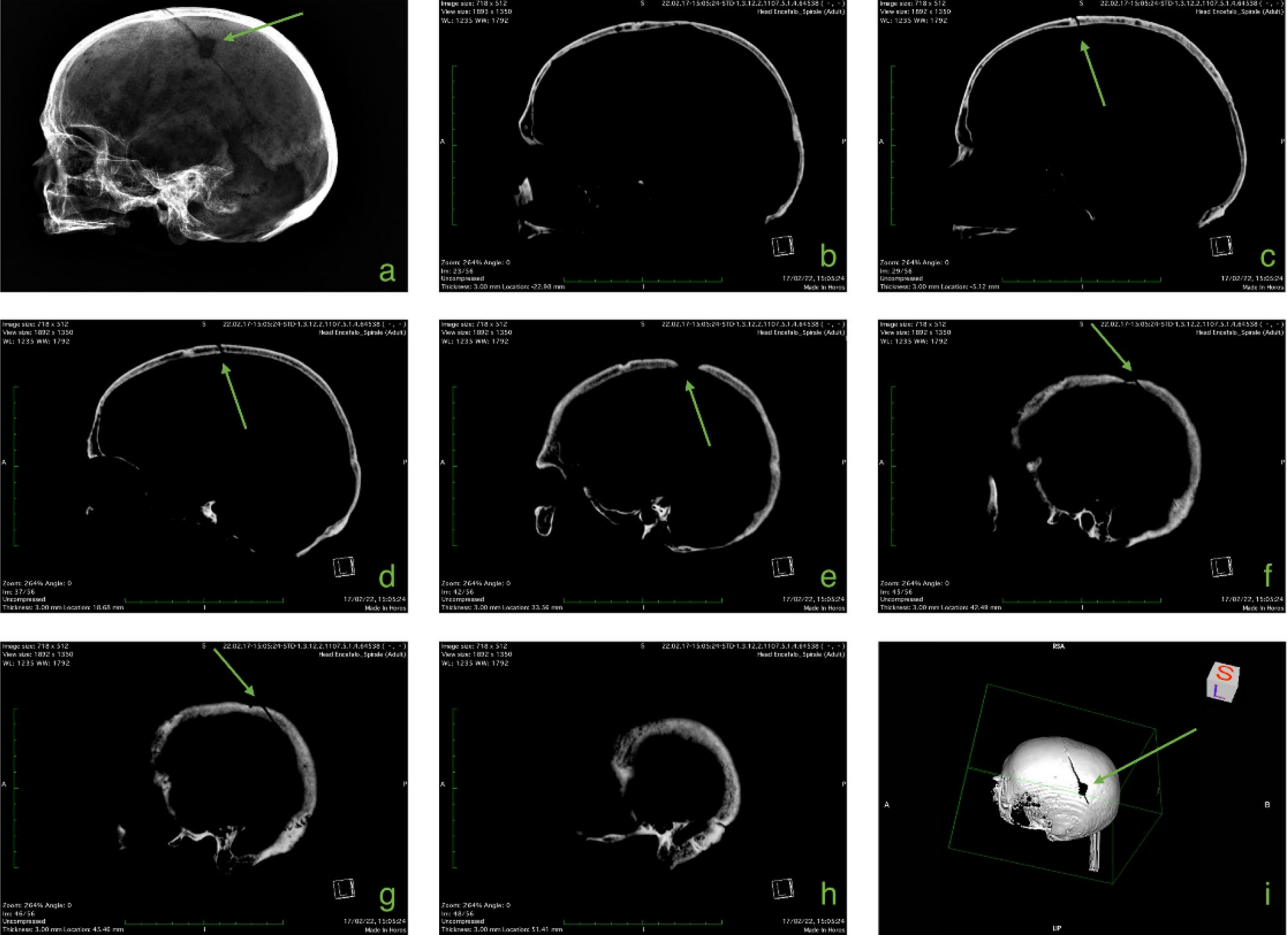
Radiological imaging of MI CG 21 O-US 4-1003. From (**a–h**), left lateral view of cranium. (**a**) X-rays imaging (green arrow: ante-mortem trauma). (**b**) CT image without signs of antemortem trauma. From (**c–g**), CT images of antemortem trauma probably followed by trepanation on left parietal bone. (**h**) CT image without signs of antemortem trauma. (**i**) Three-dimensional representation of cranium from superior lateral view of the left side.

Radiological imaging of the antemortem trauma discovered in C5 are reported in Fig. 3 through X-ray imaging, computed tomography, and 3D representation. Signs of tertiary syphilis could be evidenced on CT imaging presented in Fig. 1. The rest of the crania revealed no pathological or traumatic evidence.

### Toxicological results

Substances of toxicological interest were discovered in six of the total seventeen biological samples (35%): four out of nine preserved brain tissue (44%) and two out of eight cranial samples (25%) provided positive results, never on both brain and bone. Different active principles of opium poppy (*Papaver somniferum*) were observed (morphine, codeine, papaverine and noscapine) both in preserved brain tissue and bone samples, as reported in Table 2.

All the samples under investigation were screened with a customized inclusion list, containing the alkaloids of the medical plants listed inside the hospital pharmacopeia. The molecules under investigation were confirmed following the international standard guidelines for forensic toxicology^36^.

Therefore, the analytes were identified considering the parent ion together with characteristic fragmentation of each molecule. The signal-to-noise ratio was above 3 for all the molecules, permitting the qualitative confirmation.

Then, the confirmation was assessed via reference material (analytical standards) comparing retention time and mass spectral ion ratio.

**Figure 4:**
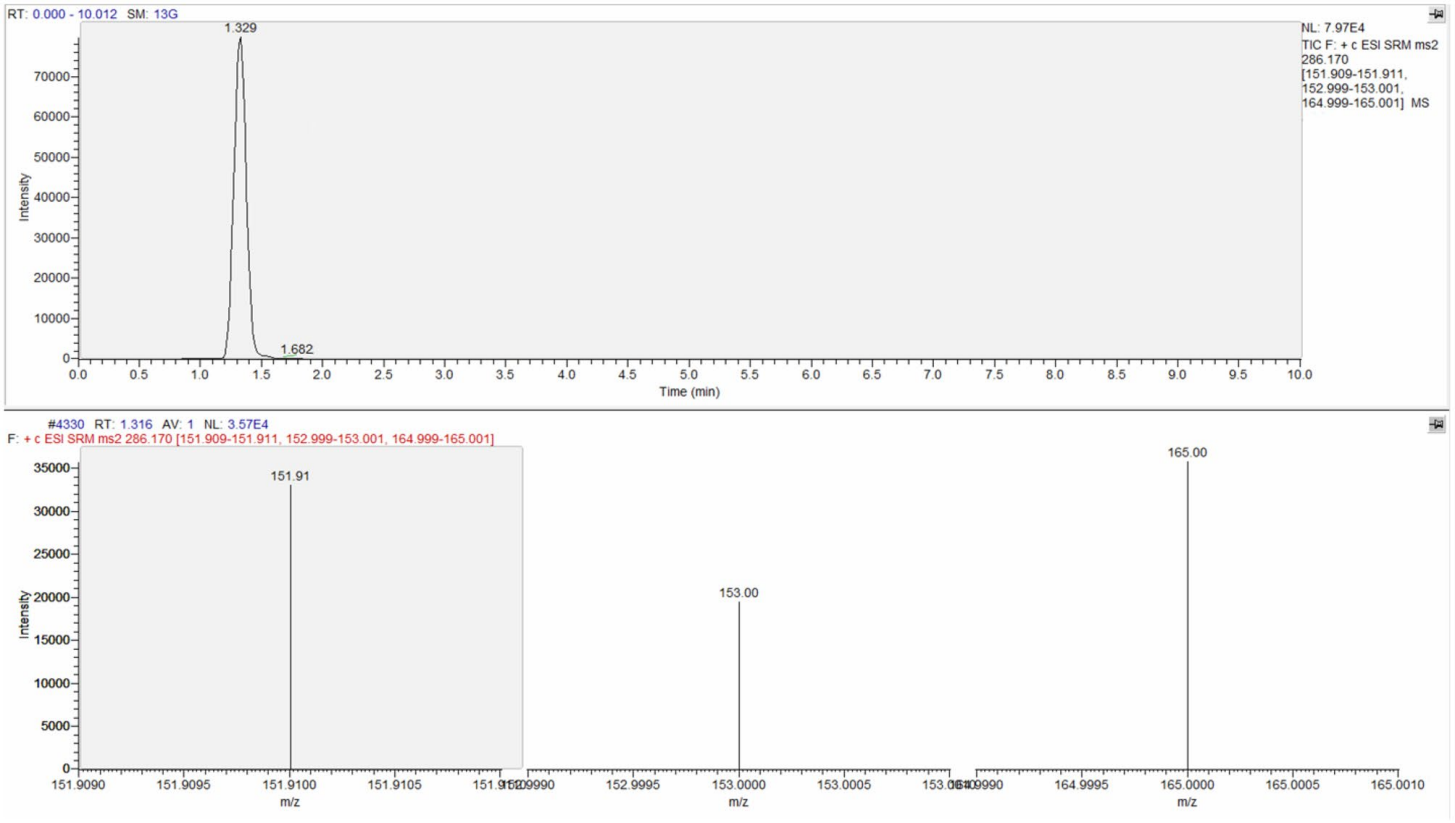
Chromatographic spectrum (upper lane) and mass spectral ion ratio (lower lane) of morphine detected in C4.

**Figure 5:**
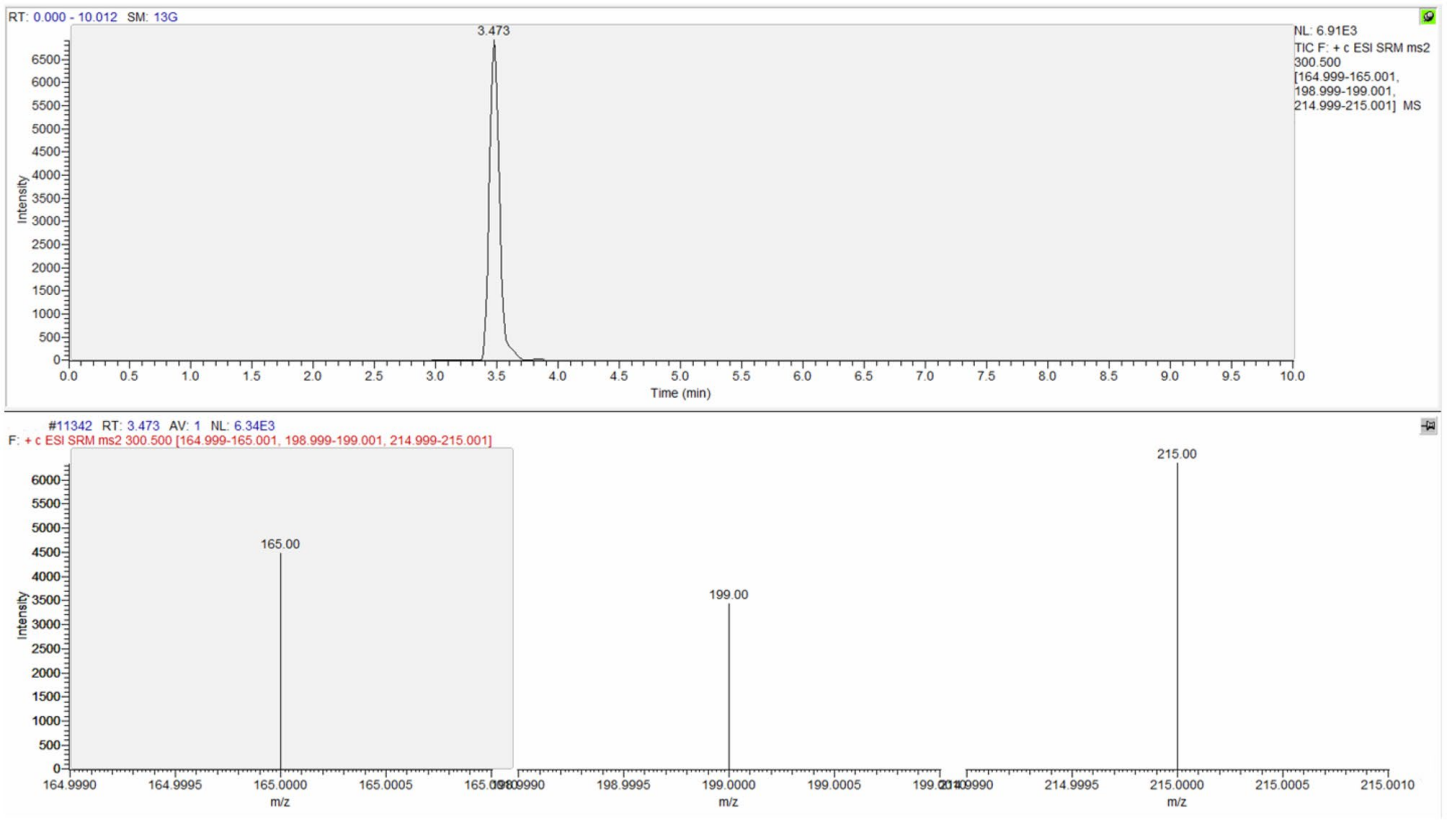
Chromatographic spectrum (upper portion) and mass spectral ion ratio (bottom portion) of codeine detected in PBT5.

**Figure 6:**
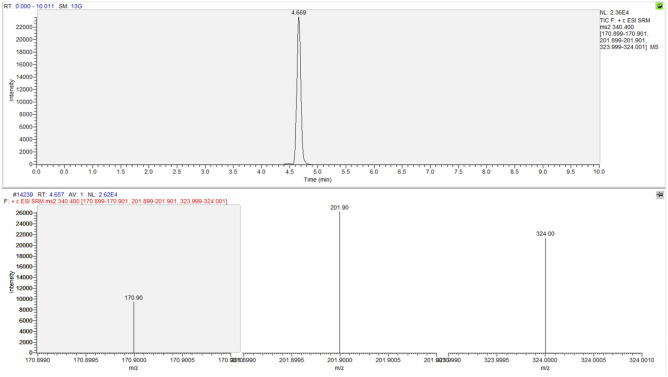
Chromatographic spectrum (upper raw) and mass spectral ion ratio (lower portion) of papaverine detected in C3.

**Figure 7:**
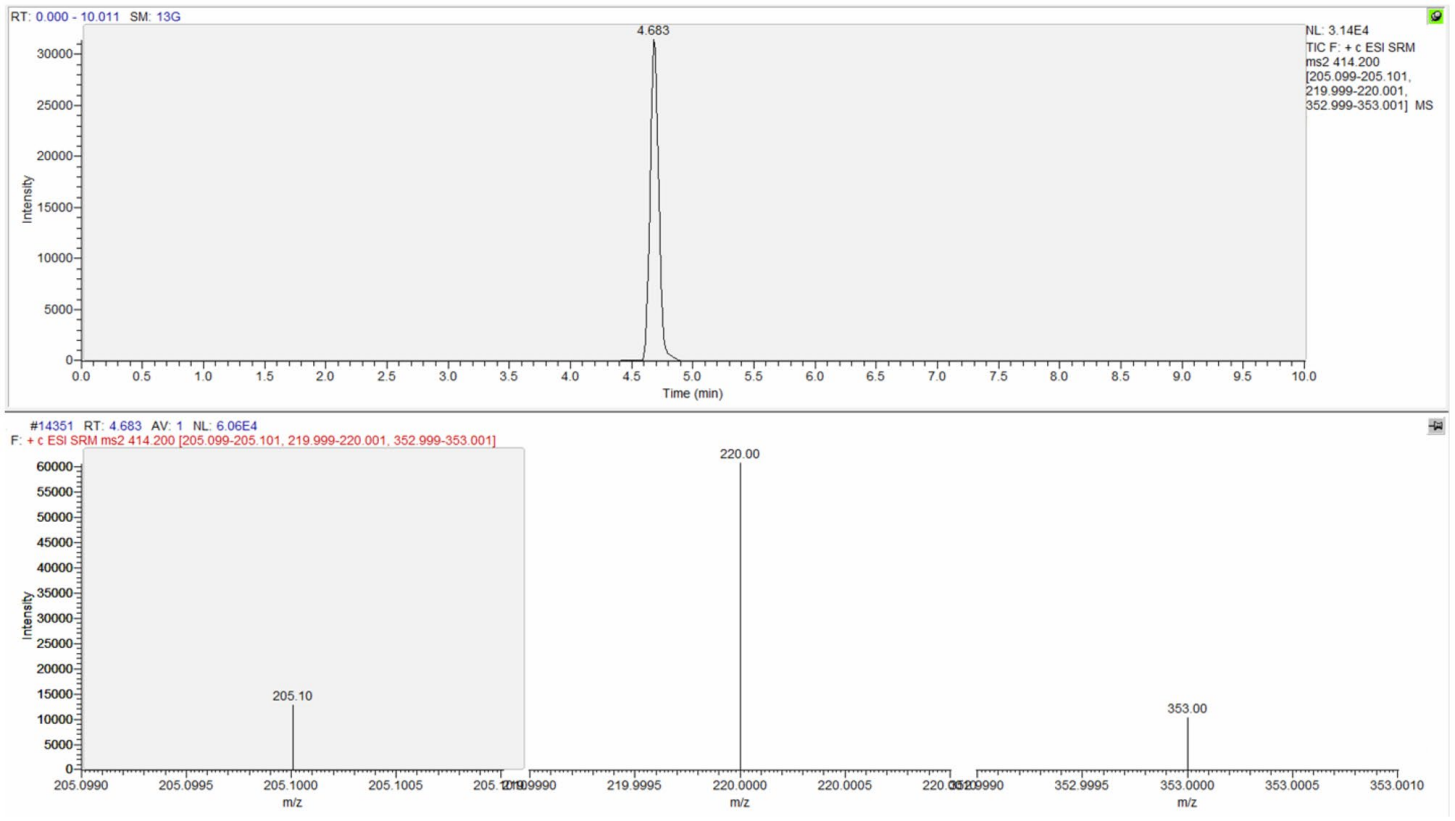
Chromatographic spectrum (upper portion) and mass spectral ion ratio (bottom raw) of noscapine detected in PBT1.

The identification criteria used for the identification of the analytes are reported in Table 3 and an example of chromatographic spectrum of each molecule are reported from Figs. 4, 5, 6 and 7.

**Table 3:** Identification criteria for molecules under investigation.

Molecule	Parent ion (m/z)	Product ion (m/z)			Retention time
Morphine	286.170	151.91	153.00	165.00	1.329
Codeine	300.500	165.00	199.00	215.00	3.473
Papaverine	340.400	170.90	201.90	324.00	4.669
Noscapine	414.200	205.10	220.00	353.00	4.683

The detection of papaverine, noscapine and codeine allowed to confirm that the plant was administered at the patients, considering that those three alkaloids can be detected only with the consumption of the *Papaver somniferum*, excluding any possible external and more recent contamination due to the heroin or morphine.

## Discussion

Toxicology applied to historical and archaeological human remains is an innovative and relatively recent discipline, referred to as archaeotoxicology. This paper aims to contribute to this field of research by searching analytical signs of the administration of medical plants in hospitalized individuals of seventeenth century Milan. The results obtained on cranial and brain samples showed the presence of molecules, namely morphine, noscapine, papaverine and codeine, which can be attributed to the intake of the plant *Papaver somniferum* by the subjects included in this study.

These results, to the best of our knowledge, constitute the first report on the detection of opium in historical and archaeological human remains. Indeed, according to the literature, this category of drugs has never been detected in soft tissues nor in bone samples. Some molecules, specifically cocaine, cotinine, and tetrahydrocannabinol were detected in ancient archaeological human remains^10,12–16,37^, even though the reliability of the interpretation of some of these works^10,38^ have been questioned^37^. Indeed, Musshoff et al*.*^37^ reported that “The findings of cocaine, nicotine, and tetrahydrocannabinol (THC) in Egyptian mummies (1000 B.C. to 400 A.D.) by Balabanova et al.^38^ were critically discussed, especially because pre-Columbian transoceanic trips are highly speculative on the grounds that two of the substances are known to be derived from American plants only: cocaine from *Erythroxylon coca* and nicotine from *Nicotiana tabacum*”*.*

However, no study has reported the detection of opium in ancient biological remains, whether they were bones, hair, nails, teeth, or preserved soft tissue, therefore we can consider this study as the first to prove the administration of opium in archaeological human remains of the Modern Age.

In the present case, the archaeotoxicological analyses performed on preserved brain tissues and cranial samples of different deceased patients of the *Ospedale Maggiore* highlighted the presence of active principles attributable and specific to opium poppy (*Papaver somniferum*), namely morphine, noscapine, papaverine and codeine, detected in both preserved brain tissues and bone samples (Table 1). Specifically, noscapine, papaverine, and codeine (active principles of *Papaver somniferum*) were noted in preserved brain tissue, whereas in addition to these molecules, morphine was also detected in bone samples. The skeletons with traces of *Papaver somniferum* belonged to three females (including two young adults), one male, and one subadult of 11–12 years (for whom sex estimation could not be performed). In one case with positive toxicological findings (no. C5), signs of ante-mortem trauma were seen on the left parietal bone (Figs. 3, 4) with loss of bone tissue and possible trepanation. This could have been responsible for chronic inflammation and pain which may have been treated with the use of the *Papaver somniferum* plant at the hospital. Indeed, opium contains a strong analgesic molecule known as morphine and the usage of opium poppy at the *Ospedale Maggiore* was confirmed by historical data: *Papaver somniferum* is present in the *Ca’ Granda* pharmacopoeia archives, showing not only that it was present in the pharmacy, but that it was also actively used as a medical treatment. Opium was listed in the apothecary's archives as early as 1558 in form of laudanum or black poppy seeds; in the inventory list of 1604 the presence of white poppy seeds, black poppy seeds, poppy syrup and thebaic opium is reported, while in 1617 the hospital also introduced laudanum patches^39^. Additionally, according to the registries preserved at the former hospital, the doctors of the Modern Age, and in particular at the Ca’ Granda, used opium reduced to dried or juice pill (*Capsulae sicca et succus capsularum inmaturarum)* and as tincture of opium (*Laudanum*), as a narcotic, analgesic, astringent, coagulant, spasmolytic and antitussive^40,41^. The findings presented in this research therefore confirm the archival data and implement our knowledge of the history of medicine in Milan.

Paleopathological signs of tertiary syphilis were found on cranium C2, yet no toxicological findings were obtained on either bone or brain samples of that individual; hence, no hypothesis regarding the administration of medical plants may be elaborated for this subject.

From a toxicological point of view, the matrices investigated (preserved brain tissue and bone samples) can be representative of two different modalities of substance administration. On the one hand, preserved brain tissues, like all organs and biological fluids, represent an administration of drugs, even in single intake, near the time of death of a subject. On the other hand, the bone tissue reveals an administration or intake of the drug over time either occasional or chronic^5^. According to these assumptions, the results obtained through toxicological analyses can clarify the events of the last hours before the death of the patients of the Ca’ Granda hospital or give some insight into the habits or previous health care of the population of Milan at the time.

Thus, from the toxicological investigation on preserved brain tissues, we can state that four individuals (PBT1, PBT6, PBT8 and PBT9) had been given derivatives obtained from opium poppy slightly before death (probably while they were at the hospital) and that the drug they had received during hospitalization may have been administered because of their therapeutic properties, given their common usage at the hospital as narcotic, analgesic, astringent, coagulant, spasmolytic and antitussive^40^. By opposition, from the bone samples that resulted positive to toxicological investigation, we can hypothesize that some opium-based preparations may have been taken by the individuals of Milan, either at the Ca’ Granda itself, on a previous occasion, or at home, to cure some occasional or chronic conditions.

## Conclusion

In this paper, we presented in the context of an interdisciplinary study between archaeotoxicology, radiology, archeology, history of medicine, anthropology, and paleopathology, the presence of opium poppy in human remains for the first time in archaeological contexts thus implementing our knowledge of the medical and health care habits in the seventeenth century. Indeed, the analyses revealed the presence of active principles (morphine, codeine, noscapine and papaverine) that can be considered derivatives of *Papaver somniferum*. This plant was present in the historical archives of the pharmacopeia of the hospital, and was prescribed as a narcotic, analgesic, astringent, coagulant, spasmolytic and antitussive agent.

## Materials and methods

### Samples of interest and brain histological confirmation

Following archaeological excavations, the human remains are now stored and curated at museum MUSA (*Museo Universitario delle Scienze Antropologiche, mediche e forensi per i Diritti Umani*—University Museum of Anthropological, medical and forensic Sciences for Human Rights) in Milan, Italy. Permissions to examine skeletal and brain remains in the present study were obtained from an agreement with the *Sopraintendenza Archeologia, Belle Arti e Paesaggio della Lombardia*, a regional institution of the Italian Ministry of Cultural Heritage, following the ethical protocol of the agreement itself.

**Figure 8:**
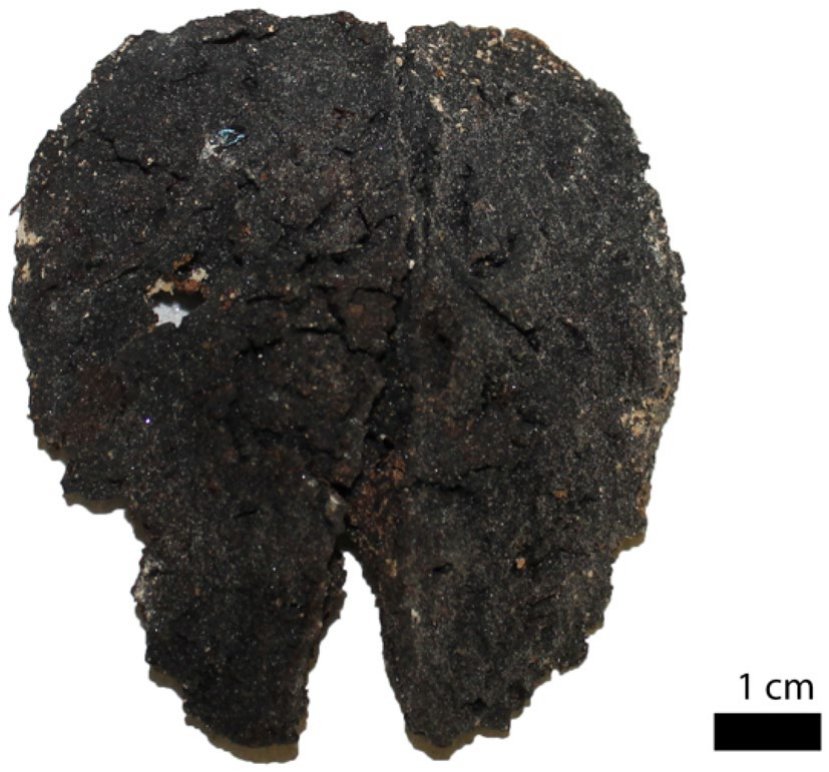
Preserved brain tissue referred to cranium MI CG 21 O-US4-155.

Eight crania were selected for study as they still contained preserved soft tissue. A ninth sample of preserved brain tissue was found during the excavation, but it was not possible to reassociate it to its cranium, therefore the soft tissue was sampled without the reassociation to the cranium of origin. Hence, nine preserved brain tissues (classified as PBT1, PBT2, PBT3, PBT4, PBT5, PBT6, PBT7, PBT8, PBT9) were collected from skeletonized crania of patients of the Ca’ Granda hospital, all belonging to Stratigraphic Unit 4 of Chamber O of the crypt (Fig. 8). These preserved brain tissues were found in situ within the vault of eight crania. All have similar consistency and appearance hence one was sampled for histological analysis.

**Figure 9:**
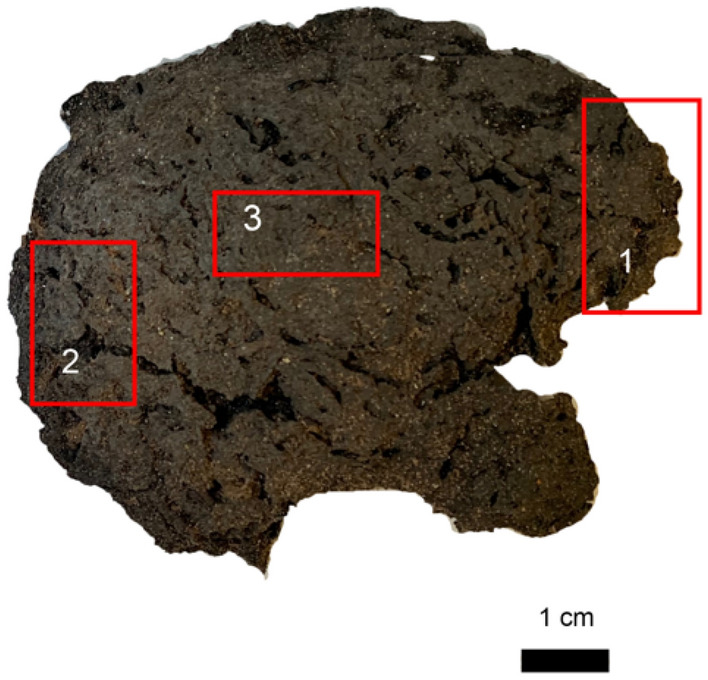
Sampling sites of brain MI CG 21 O-US4-1000 for histological investigation. Square named as 1 referred to the frontal cortex; square labeled as 2 was the occipital brain area, and square called 3 referred to parietal brain area.

Macroscopically, the mummified brain tissue presents portions of cerebral hemispheres in which gyri and grooves can be easily recognized. In cross section, a thin outer layer of gray-black color was appreciated, attributable to the cerebral cortex, and below this layer was highlighted a gray-whitish area, ascribable to white substance. The areas collected are reported in Fig. 9 and were identified as the frontal area (labeled with number 1), occipital area (labeled with number 2) and parietal cortex (named as number 3). Those three samples were collected and subsequently processed for histology following different protocols; a sample was fixed directly in 10% buffered formalin dehydrated in ethanol, according to standard post-fixative techniques, clarified and embedded in paraffin wax. One section was fixed in alcoholic 95% solution and processed, and another part was pretreated with Sandison’s solution to soften and rehydrate the tissue for 48 h, then post-fixed in formalin, embedded and cut. Following previous studies^42–44^, histological staining (H&E) on semithin sections was performed. After the histological processing, the samples were analyzed with optical microscopy^45^.

Light microscopy showed extensive decomposition artifacts, bacterial contamination and post-mortem lytic changes. The residues of cerebral parenchyma appears eosinophilic with a paucicellular component formed by a reticular matrix, crystal deposits and bacterial contamination.

Bone samples from these crania were also collected. A total of eight specimens were sampled due to the absence of the cranium associated to PBT9. Brain samples (PBT) were numbered in the same order as their respective bone; therefore, we obtained a classification from C1 to C8, with the absence of C9, as the cranium of PBT9 was missing.

#### Sample collection and preservation

The above-mentioned remains were inside chamber O of the Ca’ Granda crypt. The access to the chamber was only possible from an opening in the ceiling and the manhole had remained sealed until the first opening. At that time, the archeologists accessed the chamber equipped with personal protective equipment to perform the first site inspection. After the preliminary inspection, the first excavation campaign begun. The remains were retrieved by the archaeologists, equipped with personal protective gear and gloves, under the supervision of the toxicologists. The crania, together with their preserved brain tissue, were placed in sterilized and sealed jars and left inside the Ca’ Granda crypt to maintain the same environmental conditions of the sepulcher chamber until the time of analysis. The post-cranial bones associated to the cranium C2 were also collected together with the cranium and placed in a separate sterilized box, in order to permit the anthropologists to study the entire skeleton. The samplings performed for each investigation (toxicological, radiocarbon, and histological investigations) were performed with sterilized scalpel and handsaw.

Well-preserved brain tissue samples were collected with a scalpel. The sample site was determined based on the consistency of the samples, choosing the best preserved area, as no specific zone of sampling is suggested for forensic toxicology specimen collection in such cases^3^. For histological investigation, the section of sampling was selected based on the presence of well-preserved and well-visible encephalic convolutions, hypothesizing more preserved structures. For histological investigations three samples collected from the frontal, parietal and occipital brain area were selected.

The bone sample for the radiocarbon investigation was collected based on its weight and preservation, indeed the sample should weight about 5 g and be well-preserved with the cortical bone not affected by diagenesis or taphonomic processes. For toxicological investigations, the cranial sample was collected considering that the cranium was the only bone detected in the individuals under investigation apart from C2 that was associated with post-cranial bone samples. However, to perform a standardized sampling, we decided to collect only cranial samples, even if other bones were present (see post-cranial bone of C2). Moreover, the cranial sample can be considered a good bone matrix in forensic toxicology considering the excellent results obtained from previous studies that compared them with other bone sample sites^5,20^. The crania were cut with a sterilized handsaw on the occipital bone adjacent to the foramen magnum, and in cases where the foramen magnum was not preserved the parietal bone, in accordance with previous papers^5,20^.

### Anthropology

The crania are fragmented mostly constituted of neurocranial bones, except for C3 and C5 that were almost complete with only part of the parietal and occipital bones missing, respectively. The bones were fragile, prone to fragmentation, and often covered with a white adipocere-like staining. In spite of the commingling of the remains, C2 was anatomically associated to postcranial bones (right clavicle, right humerus and eight right ribs). Due to the absence of cranium in the case no. 9 (presence of brain tissue only), reconstruction of the biological profile was possible only in eight individuals. The subjects were assessed for age-at-death, sex and ancestry estimation using standard anthropological methods. Specifically, sex estimation was based on the evaluation of sexual dimorphic features of the cranium (in particular the anatomical points of the glabella, supraorbital margin, mastoid process, nuchal crest, and mental eminence)^27^. Age-at-death was estimated based on epiphyseal fusion, dental eruption and stage of suture closure for adult individuals^31,46–51^. When possible, biological ancestry was determined based on the correlation of eleven morphoscopic cranial traits and their frequency in reference populations to assess population affinity^33^. Pathological signs were evaluated following standard methodology^34,35,52–55^.

### Radiocarbon investigation

Radiocarbon investigation was performed to ascertain dating obtained by historical archives of the hospital. One bone sample, collected from cranium no. 2 (C2), was sent for radiocarbon dating. The results are ISO/IEC-17025:2017 accredited. All work was done at Beta Analytic Radiocarbon Dating Laboratory (Miami, Florida) in 4-in-house NEC accelerator mass spectrometry and 4 Thermo Isotope Ratio Mass Spectrometry (IRMS). The Conventional Radiocarbon Age (BP) was calculated using the Libby half-life (5568 years), is corrected for total isotopic fraction and was used for calendar calibration where applicable. The Age is rounded to the nearest 10 years and is reported as radiocarbon years before present (BP), “present” = AD 1950. Results greater than the modern reference are reported as percent modern carbon (pMC). The modern reference standard was 95% the 14C signature of NIST SRM-4990C (oxalic acid). Quoted errors are 1 sigma counting statistics. Calculated sigmas less than 30 BP on the Conventional Radiocarbon Age are conservatively rounded up to 30. δ13C values are on the material itself (not the AMS δ13C). δ13C and the δ15N values are relative to VPDB (Vienna Peedee Belemnite). References for the calendar calibrations were Ramsey^56^ and Reimer et al*.*^57^*.*

To perform the radiocarbon investigation the IRMS δ13C was − 18.8 o/oo and the IRMS δ15N was + 10.4 o/oo, with 350 ± 30 BP. The material was submitted to bone collagen extraction and the bone collagen was analyzed for the radiocarbon investigation. The pMC was 95.74 ± 0.36 and the Fraction Modern Carbon was 0.9574 ± 0.0036. The D14C was − 42.64 ± 3.58 o/oo and the Δ14C was − 50.94 ± 3.55 o/oo (1950:2022). The measured radiocarbon age (without the δ13C correction) was 250 ± 30 BP. The calibration was performed with BetaCal4.20: HPD method: INTCAL20. The ratio Carbon/Nitrogen CN: 3.3 with %C 42.47 and %N 14.84.

### Radiological imaging

All crania were imaged with x-rays to create a virtual database of the Ca’ Granda archaeological remains. In addition, imaging was performed to compare and confirm the anthropological data and to obtain radiological assessment before the sampling of bones, a disruptive procedure necessary for toxicological investigations. Furthermore, radiological investigations could reveal the presence of pathology and perhaps the reason for administration of drugs.

The crania were imaged using x-rays in frontal, lateral, superior, and inferior views, whenever possible. X-rays were performed with Poskom PXM-40BT and an X-DR L WiFi with the following technical parameters: 73–77 kV and 4 mAs. The Examion® software was used.

Two crania were also imaged with computed tomography (CT) to investigate the macroscopic signs of tertiary syphilis and antemortem trauma present on cases C3 and C6 respectively. Those crania were laid on the CT table on their skull base. CT scans were performed using a 64-slice system (Somatom Definition AS, Siemens Healthineers, Erlangen, Germany). A spiral acquisition was performed with the following parameters: 140 kVp and 40 mAs; exposure time 22.3 s; scanning length 174 mm; exposed range 170 mm; nominal single collimation width 0.6 mm; nominal total collimation width 12 mm: pitch factor 0.65. CT data were post-processed to obtain multiplanar reconstructions applying different filters using Horos v3.3.6 project, an open-source medical image viewer.

### Toxicological analyses

#### Instruments involved

A standard 12-port vacuum manifold and Bond Elut™ Certify cartridges 130 mg (Agilent) were used for SPE procedures. Samples were analyzed with a Thermo Scientific™ TSQ Fortis™ II Triple-Quadrupole Mass Spectrometer.

#### Chemicals and reagents

All the standards molecules involved in this study and the Internal Standard (IS) SKF 525-A (Proadifen hydrochloride, analytical standard, > 95%, 100 mg) as well, were purchased from Sigma-Aldrich and stored at − 20 °C. Working solutions of each molecule and IS were prepared, starting from standard solutions, and stored at − 20 °C until use. Solvents used in the extraction processes were purchased by Sigma-Aldrich (methanol) and by PanReac AppliChem ITW Reagents (buffer solution pH 9 and water for UV, HPLC, ACS).

#### Samples extraction and analyses procedure

Samples were collected from the preserved brain tissues and crania. All biological samples were powdered in a ball mill (Mixer mill MM 400, Retsch) and 0.5 g of powder were weight. One hundred ng of Internal Standard SKF 525-A (Proadifen hydrochloride), obtained from the working solutions previously prepared and correctly stored, were added to 0.5 g of each specimen. Considering the differences between the biological samples chosen, two different samples preparation were applied. For the preserved brain samples, 8 mL of pH 9 phosphate buffer solution were added, whereas cranial samples were supplemented with a water solution with EDTA at the 12.5% with decalcification effort. Both solutions obtained were agitated on a Vortex mixer (Heidolph, REAX top), placed on a rotating wheel (Falc F205) for 48 h and then centrifuged for 30 min at 3500 rpm (Thermo Scientific, Heraeus Biofuge primo centrifuge). The solutions obtained were loaded on the Bond Elut™ Certify cartridges 130 mg (Agilent) previously conditioned with 2 mL of methanol and 2 mL of pH 9 phosphate buffer. After wash out (2 mL of H_2_O, 2 mL of pH 4 buffer solution and 2 mL of MeOH), and drying of the cartridges, the samples were eluted with 2 mL of MeOH/NH_4_OH (98:2, v:v).

The eluates obtained were let dry in a vacuum rotary evaporator (Thermo Scientific, Savant SpeedVac Concentrator), then restored with 100 µL of methanol and 2 µL of these final solutions were analyzed via a Thermo Scientific™ TSQ Fortis™ II Triple-Quadrupole Mass Spectrometer. The Thermo Scientific™ TSQ Fortis™ II Triple-Quadrupole Mass Spectrometer (Thermo Scientific, San Jose, CA, U.S.A.) was associated to a HPLC system constituted by a Surveyor MS quaternary pump with degasser, Surveyor AS auto-sampler, oven with Rheodyne valve and a 20 µL loop and with a heated electrospray ionization source (HESI). The column used was a Thermo Scientific Zorbax Eclipse XDB-C18 4.6 × 50 mm, with particle size 1.8 μm reverse phase, stabilized at 35 °C and with a constant flow rate of 0.600 mL/min. Twenty mM ammonium formate in water and MeOH were the solvents that constituted the mobile phase for the analyses. The capillary and vaporization temperature were set at 330 and 280 °C respectively. The electrospray tension (with positive mode) and the positive ion spray voltage were set at 3.5 kV. Sheath gas, aux gas and sweep gas has been set at 45, 20 and 10 Arb respectively. CID gas was 1.5 mTorr, the Q1 resolution was 0.4 FWHM and Q3 resolution was 0.7 FWHM and the Resolution of power of Full Size was 70.000 FWHM. The mass range for the analyses was a range between 50 and 650 m/z. The Automatic Gain Control was set at 5 × 10^−4^ with a maximum injection time of 100 ms. The quadrupole filtered precursor ions had an isolation range of 2 m/z^58^.

### Ethics statement

Approval to conduct this research was issued by the *Sopraintendenza Archeologia, Belle Arti e Paesaggio della Lombardia*, a regional institution of the Italian Ministry of Cultural Heritage, following the ethical protocol of the agreement itself.

## Data Availability

The data underlying this article are available in the article and from the corresponding author on reasonable request.
